# Distribution of different surface modified carbon dots in pumpkin seedlings

**DOI:** 10.1038/s41598-018-26167-0

**Published:** 2018-05-22

**Authors:** Kun Qian, Huiyuan Guo, Guangcai Chen, Chuanxin Ma, Baoshan Xing

**Affiliations:** 1 College of plant protection, Southwest University, Chongqing, 400715 China; 2 Stockbridge School of Agriculture, University of Massachusetts, Amherst, MA 01003 USA; 3 Research Institute of Subtropical Forestry, Chinese Academy of Forestry, Hangzhou, Zhejiang, 311400 China; 4 Department of Analytical Chemistry, The Connecticut Agricultural Experiment Station, New Haven, Connecticut 06504 United States

## Abstract

The distribution of surface modified carbon dots (CDs) in the pumpkin seedlings was studied by visualization techniques and their potential phytotoxicity was investigated at both the physiological and biochemical levels. The average size of carbon dots was approximately 4 nm. The fluorescent peaks of bared CDs, CD-PEI and CD-PAA were between 420 nm and 500 nm, indicating CDs could emit blue and green fluorescence. Fluorescent images showed that all three types of CDs could accumulate in the pumpkin roots and translocate to the shoots, although the distribution pattern of each CDs was obviously different. At the biochemical level, the elevated antioxidant enzymes in pumpkin roots suggest that all the CDs could potentially trigger the antioxidant defense systems in pumpkin seedlings. Additionally, such alteration was greater in the roots than in the shoots. Our study represents a new perspective on CD visualization in plant tissues and provide useful information for the potential toxicity of different types of CDs to terrestrial plants, which is of importance to agricultural application.

## Introduction

Impacts of nanoparticles (NPs) on plants have been a hot issue due to the known toxicity induced by NPs, such as CuO, ZnO and TiO_2_ NPs^1–10^. Some researches have focused on the uptake and distribution of NPs with different surface modifications and surface charges in plants, which proved that different modifications could impact the NPs distribution in the plant seedlings^11,12^. However, studies on efficiency of NPs with different chemical groups entering into the plant cells were rather limited, which is important for evaluating the cytotoxicity of NPs with different surface modifications to the plants. Fluorescence labeled NPs have drawn more attention due to its visualization in plants.

Carbon dots (CDs), as novel and promising fluorescence materials, have been widely used in cell imaging, bacteria detection, printer, drug delivery *etc*.^13–15^. There are some fascinating properties such as chemical inertness, lack of blinking, low toxicity^16–18^, and excellent biocompatibility relative to traditional organic dyes and inorganic semiconductor quantum dots^19–22^.

**Figure 1:**
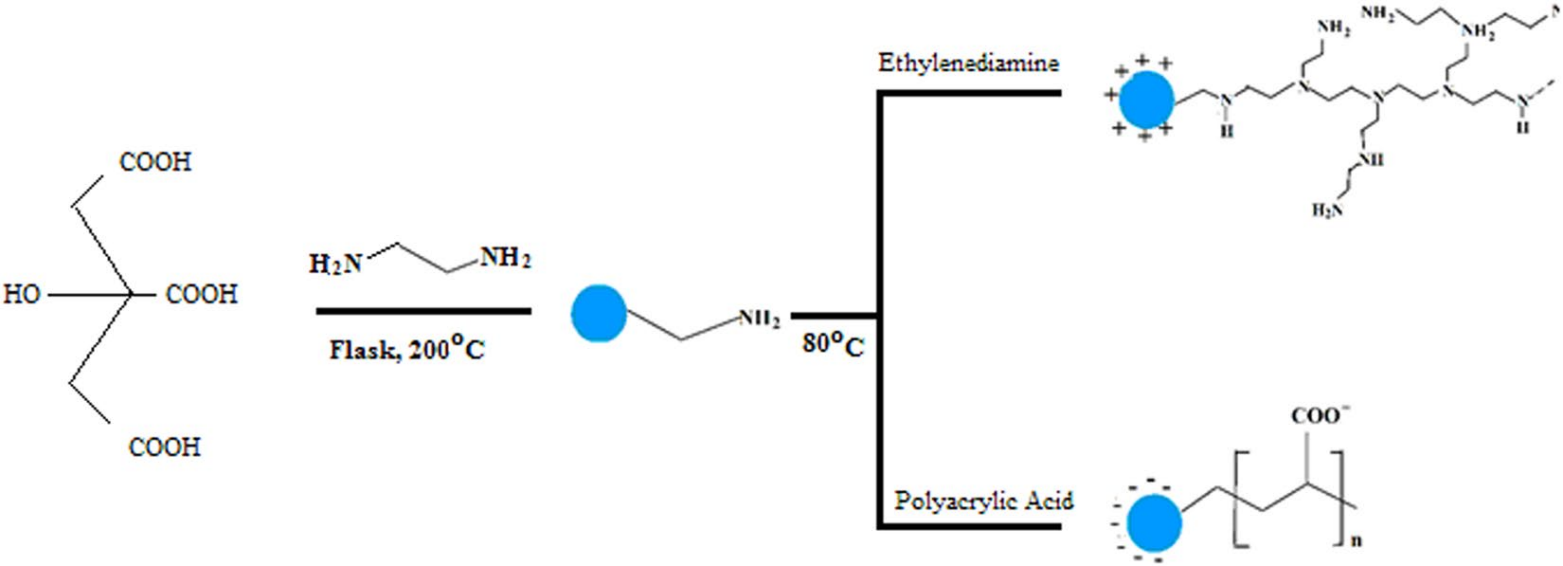
Procedures for synthesis of surface modified CDs.

The internalization efficiencies of CDs are always relatively low and the cellular fluorescence is weak^23^. Surface modification of CDs may enhance their cellular uptake by plant cells and subsequently increase the fluorescence intensity^24^. However, the cellular uptake of modified CDs may cause some unpredicted phytotoxicities to terrestrial plants. Thus, both positively and negatively charged CDs were synthesized using PEI and PAA, respectively. Illustration of the procedure for CDs  preparation with the different surface groups (CD-PAA, CDs, and CDPEI) is shown in Fig. 1. To reveal the phytotoxicity of coated and uncoated CDs, pumpkin was exposed to different concentrations (0–800 mg/L) of CDs amended full strength Hoagland’s solution over 7 days. At harvest, the fresh weight, CD distribution, MDA content as well as antioxidant enzyme activity in CD treated pumpkin were measured. Our findings provide useful information for the phytotoxicity of CDs on pumpkin seedlings and shed light on effects of surface modified CDs on the phytotoxicity of pumpkin seedlings.

## Results and Discussion

### Preparation and characterization of carbon dots

**Figure 2:**
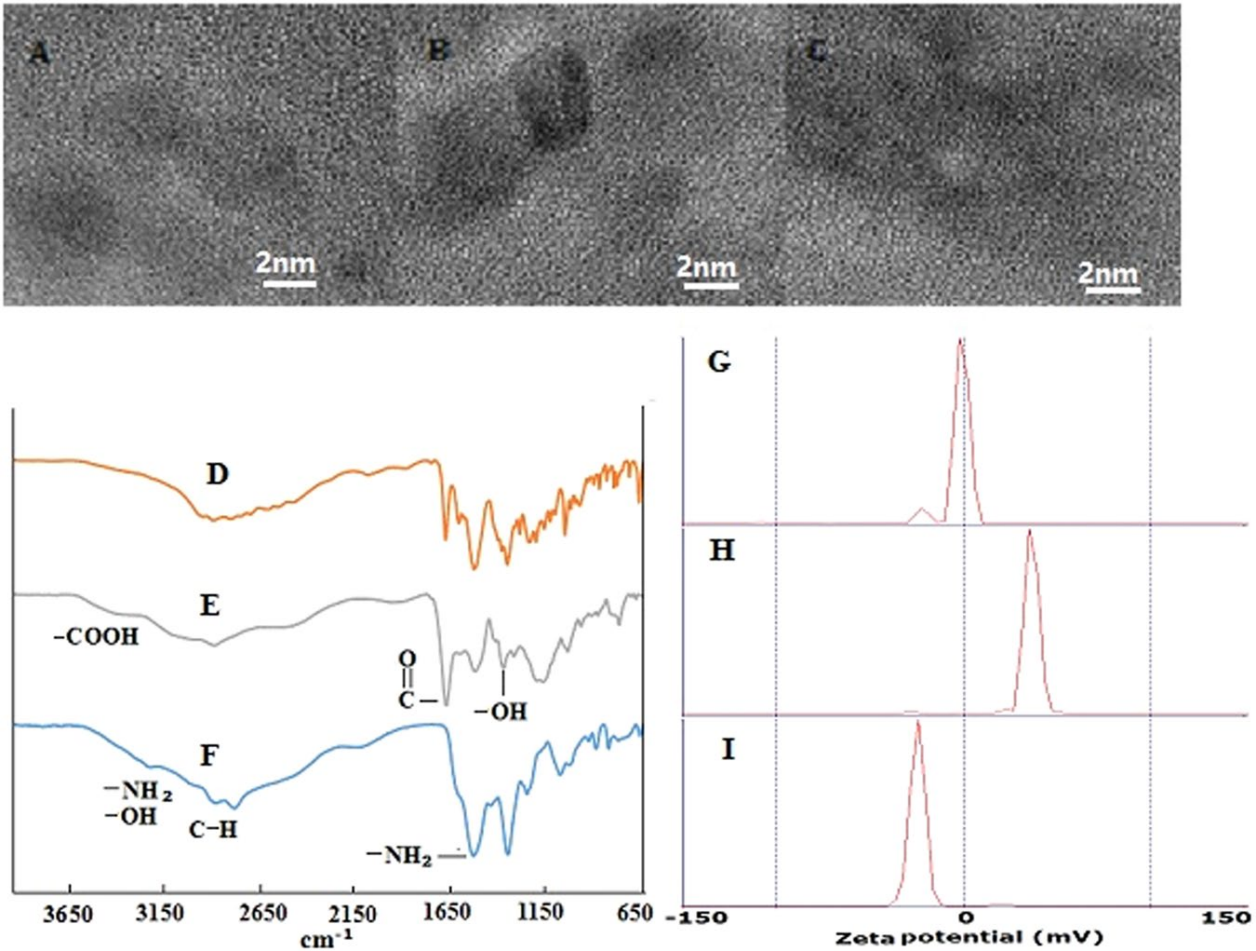
HRTEM images of (**A**) CDs, (**B**) CD-PAA, and (**C**) CD-PEI; Infrared spectra of (**D**) CDs, (**E**) CD-PAA, and (**F**) CD-PEI; Zeta potential curves of (**G**) CDs, (**H**) CD-PEI, and (**I**) CD-PAA.

Infrared spectra of hydrophobic CDs, CD-PAA, and CD-PEI are shown in Fig. 2D, E and F, respectively. The peaks of 2930 cm^−1^ and 1384 cm^−1^ are attributed to stretching and bending vibrations of C-H bonds. For CD-PAA, stretching vibrations of C=O in -COOH were clearly observed at 1701 cm^−1^. For CD-PEI, the -OH group appeared at 3435 cm^−1^.3435 cm^−1^ showed the presence of -OH. 1548 cm^−1^ derives from bending vibration of -NH_2_. The bright luminescence of CDs might be relative to amine and carboxyl group on the surface of CDs improving the fluorescence efficiency^25^.

The results of HRTEM showed that the shape of CDs were uniform, mono-dispersed sphere or hemisphere particles, and the size of CDs ranges from 2 to 6 nm, and the average size is approximately 4 nm (Fig. 2A–C). In our study, the CDs freely dispersed in water and appeared transparent without further ultrasonic dispersion, and exhibited good photo-stability and the appearance remained unchanged after storing for several months at the ambient temperature. Figure 2(G,H,I) show that zeta potential of CDs, CD-PAA, and CD-PEI were approximately −2, −25, +25 at pH 5.8.

**Figure 3:**
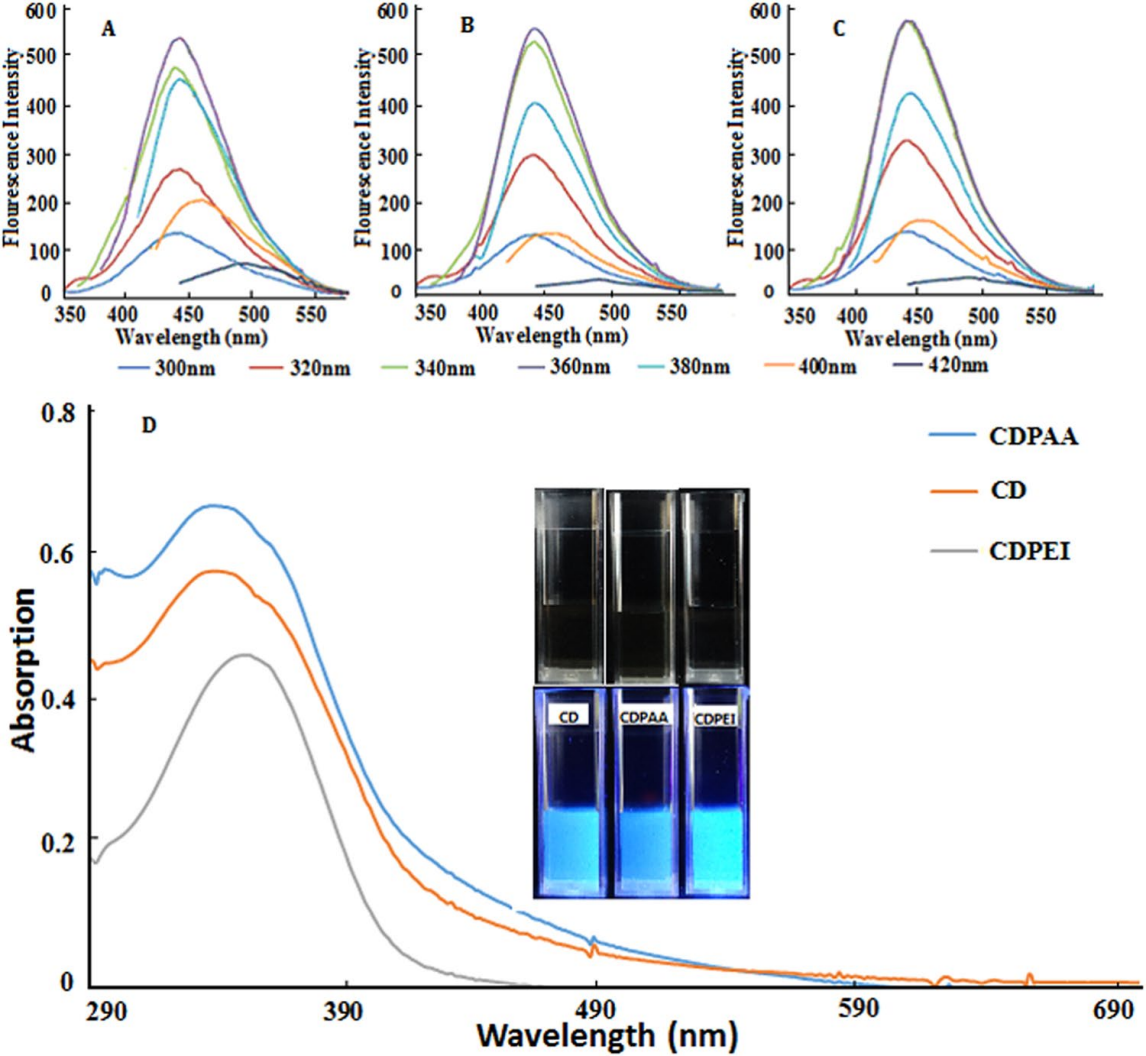
Fluorescence curves of (**A**) CD-PAA, (**B**) CDs, and (**C**) CD-PEI in excitation wavelength range of 300 nm to 420 nm; (**D**) UV spectrum of CDs, CD-PAA, and CD-PEI and the insets for the fluorescence images.

Fluorescent curves of CD-PAA (Carbon dots modified by Polyacrylic Acid), CDs, and CD-PEI (Carbon dots modified by Polyethylenimine) are shown in Fig. 3A–C, respectively. When excitation wavelengths were in the range of 300 to 420 nm, the emission peaks were from 440 nm to 500 nm. The fluorescence intensity reached the strongest with 360 nm excitation wavelengths. As excitation wavelengths shifted from 360 nm to 420 nm, fluorescence intensity was reduced. Compared with pure CDs, the PAA or PEI coated CDs showed stronger fluorescence peak.

UV-Vis spectrum of hydrophobic CDs, CD-PAA, and CD-PEI are shown in Fig. 3D. Under white light, CD solution was in light yellow. However, under the excitation at 365 nm wavelength the solution exhibited bright fluorescence light. The peak at 360 nm stands for the surface/molecule center in the UV-Vis spectrum of hydrophobic CDs.

### Effects of three types of carbon dots on pumpkin seedling growth

**Figure 4:**
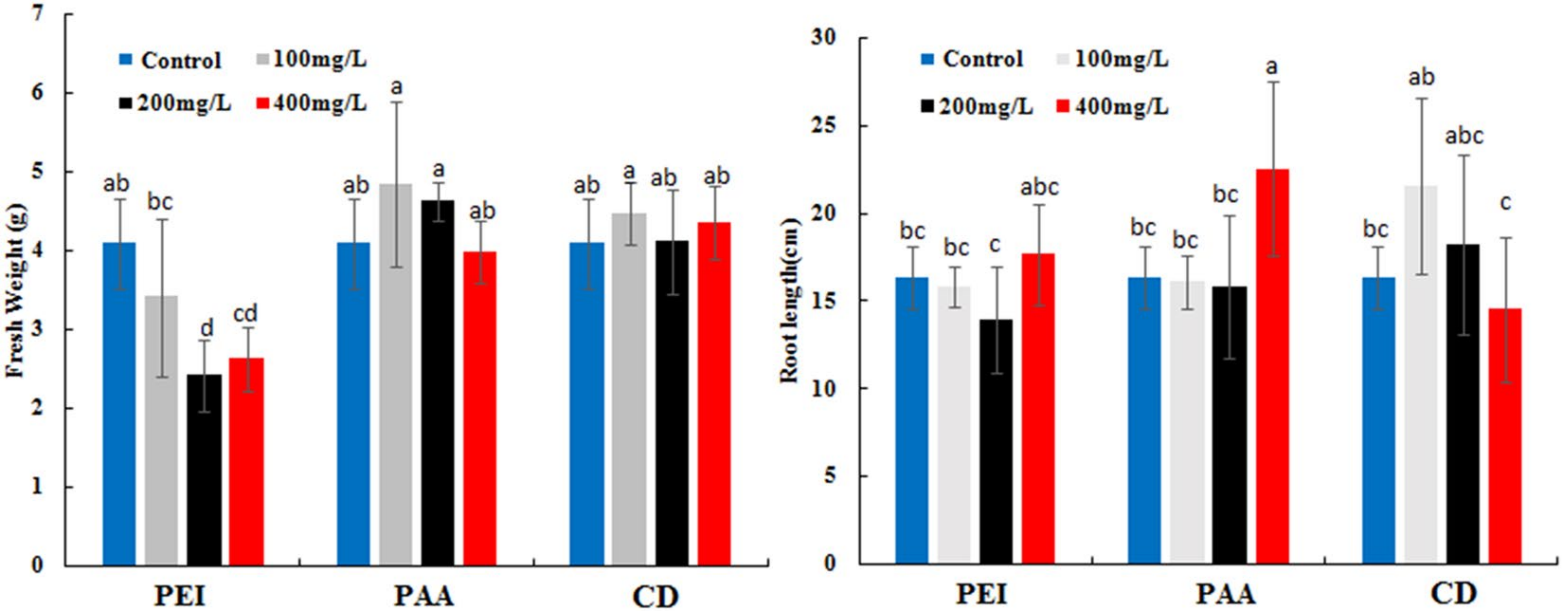
The effects of CDs with different modifications on the root length and fresh weight in the concentration range of 100–400 mg/L, ANOVA was used to evaluate the difference of fresh weight among different samples. Values of each parameter followed by different letters indicate that the data points are significantly different at p ≤ 0.05.

Root length and fresh biomass of pumpkin seedling treated with different CDs are shown in Fig. 4. Compared to the control, the fresh biomass of pumpkin seedling treated with CD-PEI decreased by about 25% significantly, however, CD-PAA and CDs had little effect on the fresh weight. For effects of CD-PAA, CD-PEI and CDs on roots length, roots length increased significantly at the highest concentration of CD-PAA compared with CD-PEI and CDs, which might be ascribed to the uptake of more nutrient elements in the seedlings together with CD-PAA due to the electrostatic absorption between the carboxy groups of CD-PAA and nutrient elements^26^.

### Distribution and fluorescence intensity of carbon dots in pumpkin seedling

**Figure 5:**
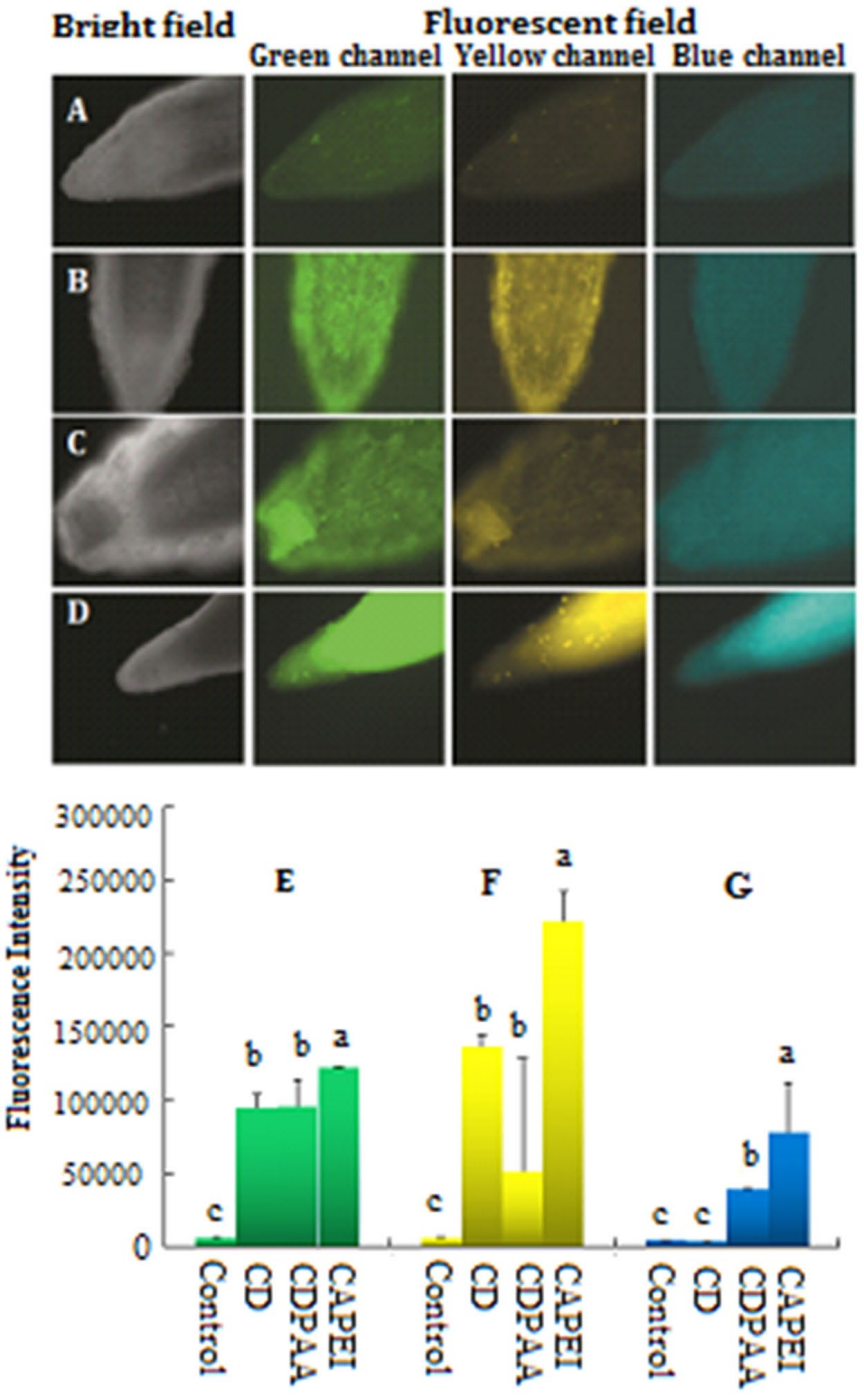
The distribution and fluorescence intensity of CDs in pumpkin root tips including (**A**) control, (**B**) CDs, (**C**) CD-PAA, (**D**) CD-PEI, (**E**) green fluorescence intensity, (**F**) yellow fluorescence intensity (**G**) blue fluorescence intensity. Values of each parameter followed by different letters indicate that the data points are significantly different at p ≤ 0.05.

As shown in Fig. 5, fluorescence intensity of CDs functions as an indicator of their distribution in pumpkin root tips. No fluorescence was observed in the control root tip, however, significant high intensity of fluorescence was evident in all the three CDs treatments with the 400 mg/L dose. In the 400 mg/L CD-PEI treatment, the fluorescence was found all over the root tips; upon exposure to 400 mg/L CDs and CD-PAA, the fluorescence was only observed in the epidermis of pumpkin roots. It could be ascribed to the effects of surface charges of CDs on CD uptake by pumpkin. The positively charged CDs (CD-PEI) enhanced the CD uptake and then resulted in elevated toxicity as determined by fresh weight as compared to bare CDs and CD-PAA.

**Figure 6:**
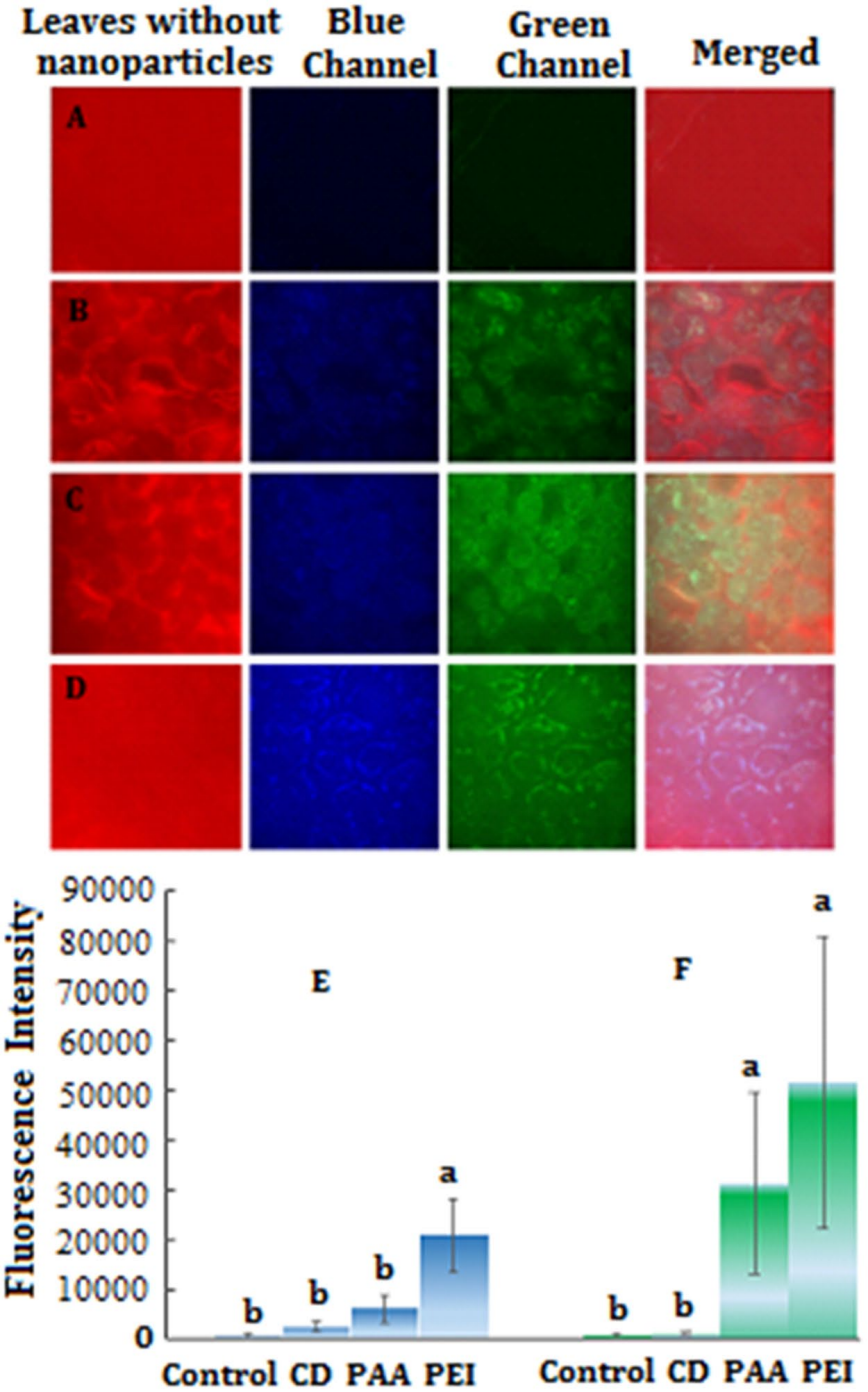
The distribution and fluorescence intensity of CDs in pumpkin leaves (**A**) control, (**B**) CDs, (**C**) CD-PAA, (**D**) CD-PEI, (**E**) blue fluorescence intensity, (**F**) green fluorescence intensity. Values of each parameter followed by different letters indicate that the data points are significantly different at p ≤ 0.05.

Figure 6 shows the distribution and fluorescence intensity of CDs (400 mg/L) in pumpkin leaves. Fluorescent images of leaves showed that the bare CDs entered into the leaf cells, while the fluorescence intensity of leaves treated with coated CDs was the weakest and leaves treated with CD-PEI had the strongest fluorescence intensity. In addition, distribution of CDs in the leaf cells was different. The whole leaves treated with CD-PEI had the strong fluorescence intensity than CDs and CD-PAA, suggesting fluorescent dye could be one of the important factors that contributed to phytotoxicity to the pumpkin seedlings.

### Lipid peroxidation, protein and antioxidant enzyme activities in pumpkin seedlings

**Figure 7:**
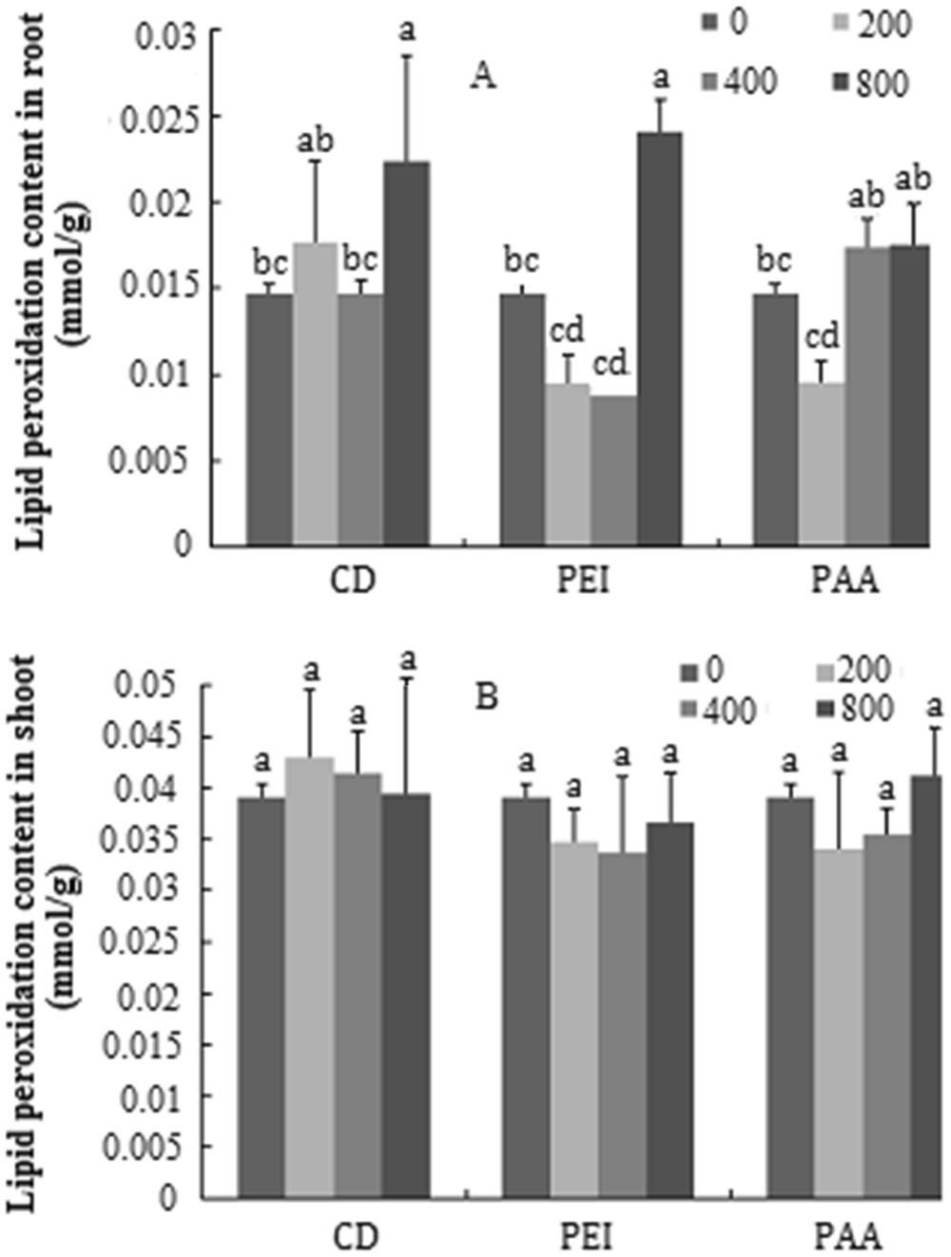
Content of lipid peroxide of pumpkin seedlings exposed to different concentrations of CDs, CD-PAA and CD-PEI (0, 200, 400 and 800 mg/L). Figure (**A**,**B**) represent content in roots and shoots, respectively. Values of each parameter followed by different letters indicate that the data points are significantly different at p ≤ 0.05.

Lipid peroxidation as measured by the malondialdehyde (MDA) content in roots and shoots of pumpkin seedlings treated with different concentrations of CDs, CD-PAA and CD-PEI is shown in Fig. 7A,B. Significant increases in the MDA content were observed in pumpkin seedlings treated with different concentrations (200–800 mg/L) of CDs, CD-PAA and CD-PEI as compared to the control. In particular, upon exposure to 800 mg/L of CD and CD-PEI, the MDA content in the roots was increased by 40% and 60% relative to the control, respectively. For the treated shoots, the MDA content had no change across all the CD treatments regardless of the exposure concentrations.

**Figure 8:**
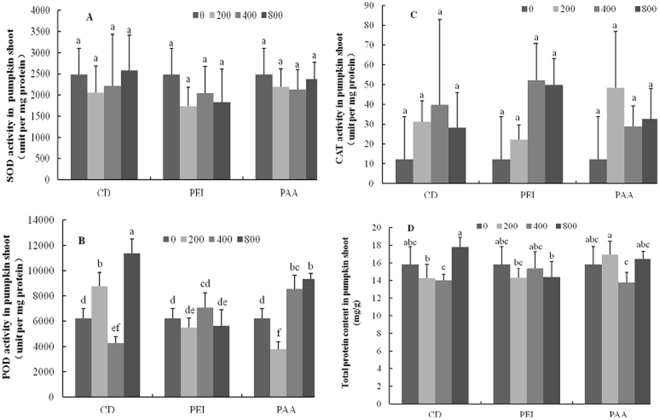
Antioxidant enzyme activities of pumpkin shoots exposed to different concentrations of CDs, CD-PAA and CD-PEI (0, 200, 400 and 800 mg/L). Figure (**A**–**D**) represent SOD, POD, CAT activities and total protein content, respectively. Values of each parameter followed by different letters indicate that the data points are significantly different at p ≤ 0.05.

**Figure 9:**
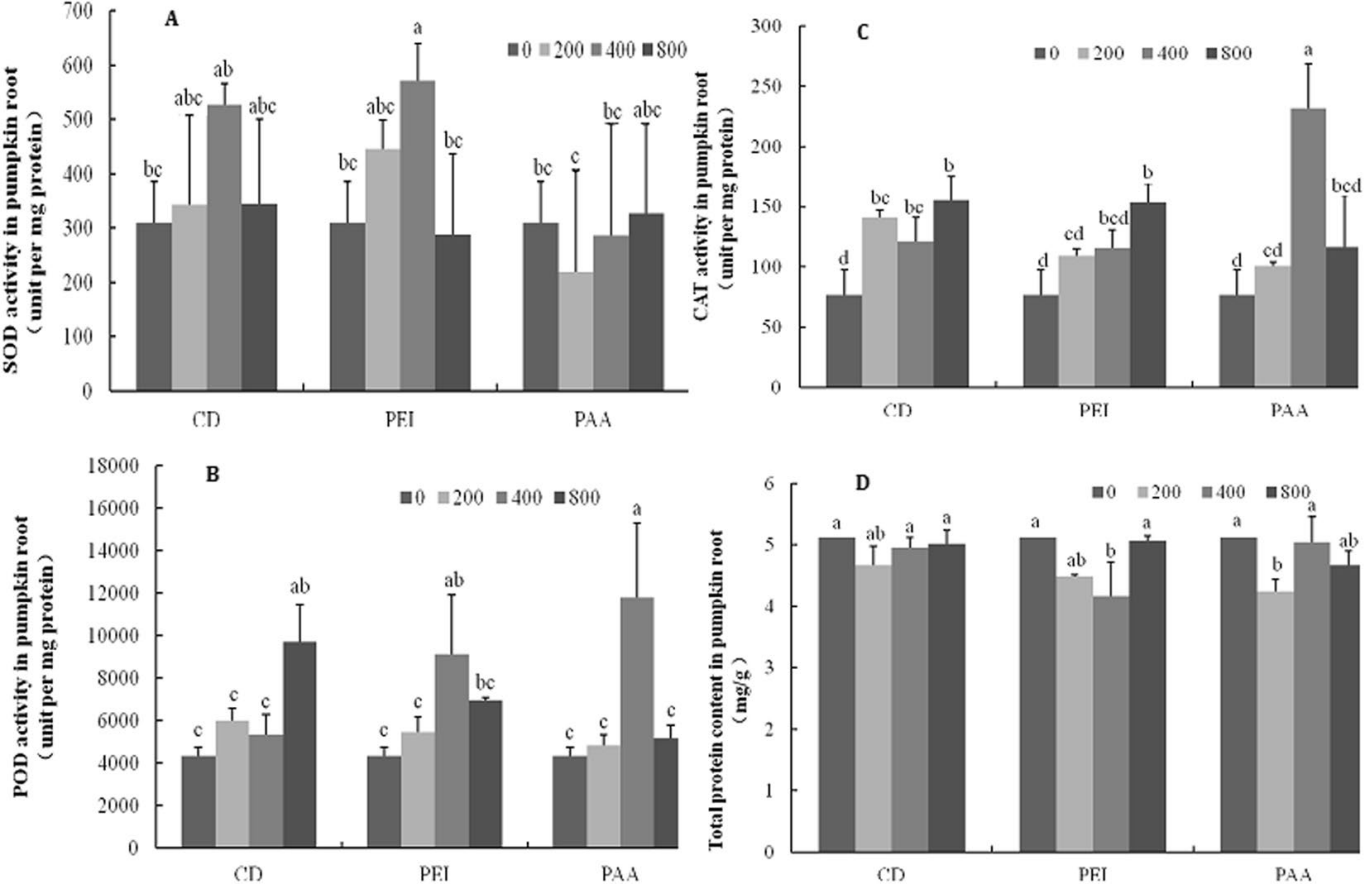
Antioxidant enzyme activities of pumpkin roots exposed to different concentrations of CDs, CD-PAA and CD-PEI (0, 200, 400 and 800 mg/L). Figure (**A**–**D**) represent SOD, POD, CAT activities and total protein content, respectively. Values of each parameter followed by different letters indicate that the data points are significantly different at p ≤ 0.05.

The activity of antioxidant enzymes (SOD, POD, and CAT) and the total protein content in CD treated pumpkin shoots were measured (Fig. 8). Upon exposure to different concentrations of CDs, the activities of the shoot SOD and CAT had no change as compared to the control. However, 400 and 800 mg/L CD-PAA notably elevated the POD activity by 50% relative to the control. Similar result was evident in 200 and 800 mg/L CDs treated shoots. No change was found among all the CD-PEI treatments. In addition, the total protein content in CDs treated pumpkin shoots and roots had no change. The SOD activity in all types of CDs treated pumpkin roots was similar to the control, except 400 mg/L CD-PEI, where the SOD activity was approximately 50% higher relative to the control (Fig. 9). Exposure to CDs significantly elevated the root CAT activity by more than 40% relative to the control. However, no difference on the CAT activity was found in the modified CD treatments, except 800 mg/L CD-PEI and 400 mg/L CD-PAA, where the CAT activity was significantly increased. Interestingly, 800 mg/L bared CDs elevated the POD activity by more than 100% and both 400 mg/L surface modified CDs was also increased by approximately 100% relative to the control. Taken together, exposure to three types of CDs had more impacts on the pumpkin roots than the shoots^27^.

## Conclusions

The cellular uptake of different surface modified CDs was studied using pumpkin as a model plant. The three types of CDs with different modifications were prepared and characterized by TEM and IR. The average size is approximately 4 nm. Florescent images of the root tips demonstrated that all CDs could translocate to the pumpkin roots with distinctive distribution. PEI and PAA coated CDs yielded high amounts of fluorescent intensity in pumpkin seedlings. In comparison with bared CDs and CDs-PAA, exposure to 400 and 800 mg/L CDs-PEI significantly decreased the total fresh weight. Although no physiological alteration was evident in other two CDs, the activities of antioxidant enzymes in pumpkin seedlings were changed, suggesting the defense mechanisms were triggered at the biochemical level. Our study provides a new perspective on characterizing CD uptake by and their potential phytotoxicity to terrestrial plants.

## Method

### Preparation and characterization of carbon dots

Citric acid was used as a carbon sources for carbon dots preparation using hydro-thermal method^28^ (Fig. 1). Citric acid (1.0507 g, Sigma Aldrich) and ethylenediamine (335 μL, Sigma Aldrich) was dissolved in DI-water (10 mL). Then the solution was transferred to a flask (50 mL) and heated at 200 °C overnight. After the reaction, the reactors were cooled to room temperature naturally. The production yield was ca. 50%. 1 mL of 10% polyacrylic acid (PAA, Sigma Aldrich) and polyethylenimine (PEI, Sigma Aldrich) solution were added into the CDs solution respectively and heated at 80 °C for 4 hour to obtain CD-PAA and CD-PEI. All the products were subjected to dialysis in a dialysis bag and dried by a vacuum freeze dryer.

The fluorescence performances of all three CDs were measured by fluorescence spectrometer (Eclipse 80i). The particle size, zeta potential, and structure of carbon dots nanoparticles were characterized by a dynamic light scattering (DLS, Zetasizer Nano ZS90) and a high resolution Transmission Electron Microscopy (HRTEM, Zeiss Libra 200). The chemical groups on the surface of each CDs were characterized by an infrared Spectroscopy (IR, PE Spectrum 100).

### Hydroponic Culture

Surface sterilized pumpkin seeds were grown in moist vermiculite for 2 weeks. Uniform seedlings were selected and transplanted to 100 mL beakers containing 100 mL of full strength Hoagland’s solution (pH 5.8, Sigma Aldrich). All containers were wrapped with aluminum foils to avoid light. The seedling roots were submerged into the nutrient solution and allowed to acclimatize for one week prior to CD exposure.

### Observation of carbon dots in pumpkin cells

Root tips and leaves tissues were cut into pieces and mounted onto a slide and observed under a fluorescence microscope and a confocal laser scanning microscopy (OLYMPUS FV1000), respectively, to confirm whether the CDs could accumulate and distribute in plant cells. Fluorescence intensity of CDs with different modifications in plant tissues were measured by Image J. software.

### Assays for antioxidant enzyme activities and malondialdehyde contents

Fresh pumpkin shoots and roots were separately ground into a fine powder in liquid nitrogen. The activities of the antioxidant enzymes, SOD, CAT, and POD, were measured as described in Ma *et al*.^29^. The Bradford method was used to determine the protein concentration in pumpkin tissues.

Lipid peroxidation was determined in terms of malondialdehyde (MDA) content using the thiobarbituric acid (TBA) method^30^.

### Statistical analyses

A one-way analysis of variance (One-way ANOVA) followed by Duncan’s multiple comparison test was used to determine statistical significance of each parameter across treatments.
